# 4-Bromo-2-[(*E*)-(2-fluoro-5-nitro­phenyl)iminometh­yl]phenol

**DOI:** 10.1107/S1600536812050696

**Published:** 2012-12-19

**Authors:** Shaaban K. Mohamed, Mehmet Akkurt, Peter N. Horton, Antar A. Abdelhamid, Adel A. Marzouk

**Affiliations:** a Chemistry and Environmental Division, Manchester Metropolitan University, Manchester M1 5GD, England; b Chemistry Department, Faculty of Science, Minia University, El-Minia, Egypt; c Department of Physics, Faculty of Sciences, Erciyes University, 38039 Kayseri, Turkey; d School of Chemistry, University of Southampton, Highfield, Southampton SO17 1BJ, England; e Pharmaceutical Chemistry Department, Faculty of Pharmacy, Al AzharUniversity, Egypt

## Abstract

The mol­ecular conformation of the title compound, C_13_H_8_BrFN_2_O_3_, is essentially planar, with maximum deviations of 0.076 (1) and −0.080 (2) Å for the O atoms of the NO_2_ group. The mol­ecular conformation is stabilized by an intra­molecular O—H⋯N hydrogen bond, forming an *S*(6) ring motif. In the crystal, pairs of mol­ecules are linked *via* two pairs of C—H⋯O hydrogen bonds, forming inversion dimers that enclose *R* ^2^_2_(7)*R* ^2^_2_(10)*R* ^2^_2_(7) ring motifs.

## Related literature

For the synthesis and biological activity of azomethines, see: Przybylski *et al.* (2009 ▶); Kalaivani *et al.* (2012 ▶); Blair *et al.* (2000 ▶). For the synthesis of fluorinated azomethines, see: Mohamed *et al.* (2012 ▶). For hydrogen-bond motifs, see: Bernstein *et al.* (1995 ▶). For standard bond lengths, see: Allen *et al.* (1987 ▶).
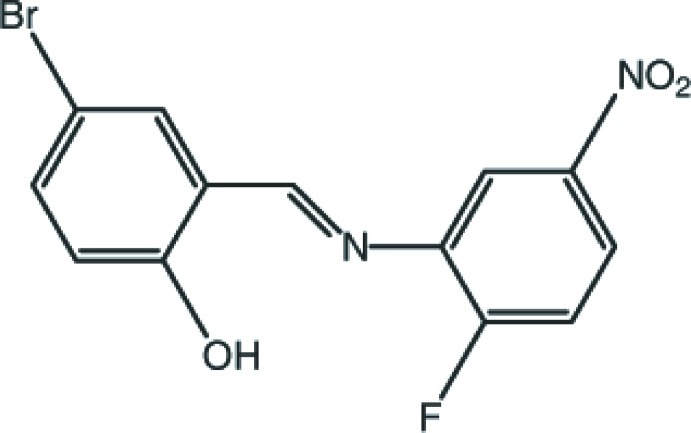


## Experimental

### 

#### Crystal data

C_13_H_8_BrFN_2_O_3_
*M* *_r_* = 339.11
Monoclinic, 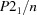

*a* = 4.5082 (9) Å
*b* = 19.815 (4) Å
*c* = 13.853 (3) Å
β = 95.484 (5)°
*V* = 1231.8 (4) Å^3^
*Z* = 4
Mo *K*α radiation
μ = 3.36 mm^−1^
*T* = 100 K
0.24 × 0.04 × 0.03 mm

#### Data collection

Rigaku AFC12 (Right) diffractometer
Absorption correction: multi-scan (*CrystalClear-SM Expert*; Rigaku, 2012 ▶) *T*_min_ = 0.500, *T*_max_ = 0.906
8107 measured reflections
2811 independent reflections
2633 reflections with *I* > 2σ(*I*)
*R*_int_ = 0.023

#### Refinement

*R*[*F* ^2^ > 2σ(*F* ^2^)] = 0.025
*wR*(*F* ^2^) = 0.059
*S* = 1.05
2811 reflections
182 parameters
H-atom parameters constrained
Δρ_max_ = 0.52 e Å^−3^
Δρ_min_ = −0.54 e Å^−3^

### 

Data collection: *CrystalClear-SM Expert* (Rigaku, 2012 ▶); cell refinement: *CrystalClear-SM Expert*; data reduction: *CrystalClear-SM Expert*; program(s) used to solve structure: *SHELXS97* (Sheldrick, 2008 ▶); program(s) used to refine structure: *SHELXL97* (Sheldrick, 2008 ▶); molecular graphics: *ORTEP-3 for Windows* (Farrugia, 2012 ▶); software used to prepare material for publication: *WinGX* (Farrugia, 2012 ▶) and *PLATON* (Spek, 2009 ▶).

## Supplementary Material

Crystal structure: contains datablock(s) global, I. DOI: 10.1107/S1600536812050696/xu5665sup1.cif

Structure factors: contains datablock(s) I. DOI: 10.1107/S1600536812050696/xu5665Isup2.hkl

Supplementary material file. DOI: 10.1107/S1600536812050696/xu5665Isup3.cml

Additional supplementary materials:  crystallographic information; 3D view; checkCIF report

## Figures and Tables

**Table 1:** Hydrogen-bond geometry (Å, °)

*D*—H⋯*A*	*D*—H	H⋯*A*	*D*⋯*A*	*D*—H⋯*A*
O1—H1⋯N1	0.84	1.86	2.601 (2)	146
C7—H7⋯O3^i^	0.95	2.45	3.399 (3)	173
C13—H13⋯O3^i^	0.95	2.48	3.430 (3)	173

Symmetry code: (i) 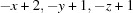.

**Fig. 1.**
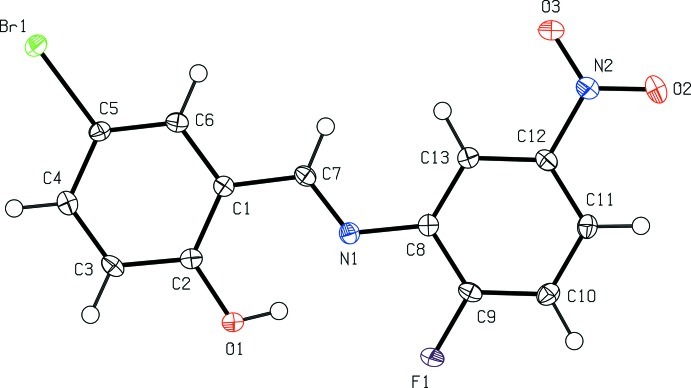
The molecular structure of (I) with ellipsoids drawn at the 50% probability level.

**Fig. 2.**
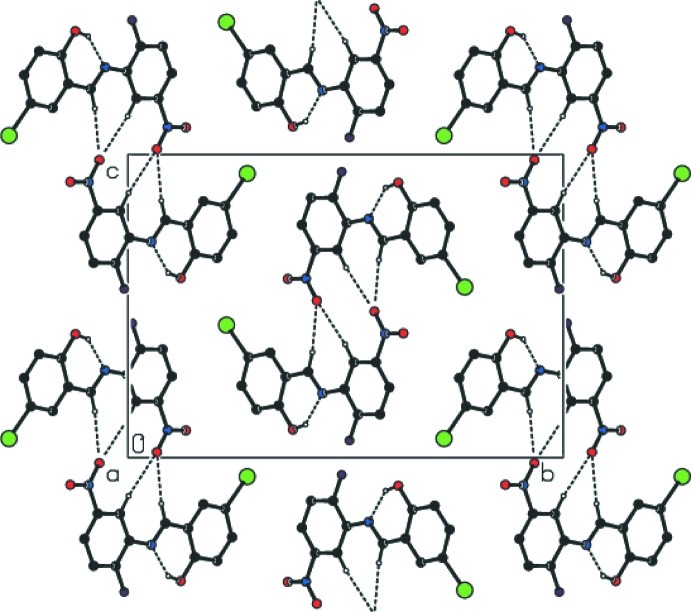
Crystal packing of (I) viewed along the a axis. The hydrogen atoms not involved in the hydrogen bonds have been omitted for clarity.

## supplementary crystallographic information

### Comment

Schiff bases have been shown to exhibit a broad range of biological activities, including antifungal, antibacterial, antimalarial, antiproliferative, anti-inflammatory, antiviral, and antipyretic properties (Przybylski *et al*., 2009; Kalaivani *et al.*, 2012). Among such compounds, the fluorinated Schiff's bases were considered to possess a distingushed biological activity due to the dramatic affect of fluorine atom on the metabolism and distribution of drug molecules in the body (Blair *et al*., 2000). Further to our on going study on synthesis of bioactive fluorinated compounds (Mohamed *et al.*, 2012) we herein report the synthesis and crystal structure of a new fluorinated azomethine derivative.

In the title compound (I), (Fig. 1), the molecular conformation is essentially planar, with maxium deviations of 0.076 (1) and -0.080 (2) Å, respectively, for O2 and O3. The C1–C7–N1–C8 torsion angle is 179.92 (16)°. The bond lengths and angles in (I) are within the normal range (Allen *et al.*, 1987).

Molecular conformation is stabilized by O—H···N hydrogen bond (Table 1), forming an S(6) ring motif. In the crystal, the pairs of molecules are linked by C—H···O interactions (Table 1, Fig. 2), generating *R*^2^_2_(7)*R*^2^_2_(10)*R*^2^_2_(7) ring motifs (Bernstein *et al.*, 1995) along the [001] direction.

### Experimental

A mixture of 1 mmol (156 mg) 2-fluoro-5-nitroaniline and 1 mmol (201 mg) 5-bromo-2-hydroxybenzaldehyde in 50 ml e thanol was heated at 350 K and monitored by TLC till completion after 12 h. A mass solid product was deposited once the reaction mixture was allowed to cool at room temperature. The crude product was filtered dried under vacuum and washed by ethanol. Pure yellow rods (m.p. 465 K) suitable for X-ray diffraction were obtained in an excellent yield (92%) by crystallization of crude product from ethanol.

### Refinement

H atoms were positioned geometrically and refined using a riding model, with O—H = 0.84 Å, C—H = 0.95 Å, and with *U*_iso_(H) = 1.5*U*_eq_(O) for hydroxyl and *U*_iso_(H) = 1.2 *U*_eq_(C) for the other H atoms.

### Crystal data

**Table:**

C_13_H_8_BrFN_2_O_3_	*F*(000) = 672
*M**_r_* = 339.11	*D*_x_ = 1.829 Mg m^−^^3^
Monoclinic, *P*2_1_/*n*	Mo *K*α radiation, λ = 0.71075 Å
Hall symbol: -P 2yn	Cell parameters from 4566 reflections
*a* = 4.5082 (9) Å	θ = 2.5–31.2°
*b* = 19.815 (4) Å	µ = 3.36 mm^−^^1^
*c* = 13.853 (3) Å	*T* = 100 K
β = 95.484 (5)°	Rod, yellow
*V* = 1231.8 (4) Å^3^	0.24 × 0.04 × 0.03 mm
*Z* = 4	

### Data collection

**Table:**

Rigaku AFC12 (Right) diffractometer	2811 independent reflections
Radiation source: Rotating Anode	2633 reflections with *I* > 2σ(*I*)
Detector resolution: 28.5714 pixels mm^-1^	*R*_int_ = 0.023
profile data from ω–scans	θ_max_ = 27.5°, θ_min_ = 3.0°
Absorption correction: multi-scan (*CrystalClear-SM Expert*; Rigaku, 2012)	*h* = −5→5
*T*_min_ = 0.500, *T*_max_ = 0.906	*k* = −25→24
8107 measured reflections	*l* = −17→14

### Refinement

**Table:**

Refinement on *F*^2^	Primary atom site location: structure-invariant direct methods
Least-squares matrix: full	Secondary atom site location: difference Fourier map
*R*[*F*^2^ > 2σ(*F*^2^)] = 0.025	Hydrogen site location: inferred from neighbouring sites
*wR*(*F*^2^) = 0.059	H-atom parameters constrained
*S* = 1.05	*w* = 1/[σ^2^(*F*_o_^2^) + (0.0255*P*)^2^ + 1.1447*P*] where *P* = (*F*_o_^2^ + 2*F*_c_^2^)/3
2811 reflections	(Δ/σ)_max_ = 0.001
182 parameters	Δρ_max_ = 0.52 e Å^−^^3^
0 restraints	Δρ_min_ = −0.54 e Å^−^^3^

### Special details

**Table:**

Geometry. Bond distances, angles *etc*. have been calculated using the rounded fractional coordinates. All su's are estimated from the variances of the (full) variance-covariance matrix. The cell e.s.d.'s are taken into account in the estimation of distances, angles and torsion angles
Refinement. Refinement on *F*^2^ for ALL reflections except those flagged by the user for potential systematic errors. Weighted *R*-factors *wR* and all goodnesses of fit *S* are based on *F*^2^, conventional *R*-factors *R* are based on *F*, with *F* set to zero for negative *F*^2^. The observed criterion of *F*^2^ > σ(*F*^2^) is used only for calculating -*R*-factor-obs *etc*. and is not relevant to the choice of reflections for refinement. *R*-factors based on *F*^2^ are statistically about twice as large as those based on *F*, and *R*-factors based on ALL data will be even larger.

### Fractional atomic coordinates and isotropic or equivalent isotropic displacement parameters (Å^2^)

**Table:**

	*x*	*y*	*z*	*U*_iso_*/*U*_eq_	
Br1	−0.15532 (4)	0.22619 (1)	0.43736 (1)	0.0188 (1)	
F1	0.7510 (2)	0.50903 (6)	0.05672 (7)	0.0202 (3)	
O1	0.2171 (3)	0.37891 (7)	0.09274 (9)	0.0171 (4)	
O2	1.5039 (3)	0.63282 (8)	0.41030 (11)	0.0290 (4)	
O3	1.2227 (5)	0.56614 (11)	0.48212 (12)	0.0614 (8)	
N1	0.5847 (3)	0.44689 (7)	0.21201 (11)	0.0140 (4)	
N2	1.3057 (4)	0.59101 (9)	0.40914 (12)	0.0227 (5)	
C1	0.2662 (4)	0.36222 (8)	0.26646 (12)	0.0123 (5)	
C2	0.1398 (4)	0.34652 (9)	0.17204 (12)	0.0135 (5)	
C3	−0.0742 (4)	0.29535 (9)	0.15895 (13)	0.0157 (5)	
C4	−0.1635 (4)	0.26099 (9)	0.23746 (13)	0.0159 (5)	
C5	−0.0389 (4)	0.27669 (9)	0.33084 (12)	0.0140 (5)	
C6	0.1728 (4)	0.32683 (9)	0.34603 (12)	0.0145 (5)	
C7	0.4936 (4)	0.41399 (9)	0.28338 (13)	0.0138 (5)	
C8	0.8050 (4)	0.49730 (9)	0.22592 (13)	0.0136 (5)	
C9	0.8877 (4)	0.52873 (9)	0.14215 (12)	0.0150 (5)	
C10	1.0992 (4)	0.57931 (9)	0.14290 (13)	0.0165 (5)	
C11	1.2365 (4)	0.60049 (9)	0.23159 (13)	0.0157 (5)	
C12	1.1566 (4)	0.56969 (9)	0.31484 (13)	0.0156 (5)	
C13	0.9451 (4)	0.51880 (9)	0.31435 (13)	0.0154 (5)	
H1	0.34830	0.40790	0.10930	0.0260*	
H3	−0.15830	0.28430	0.09540	0.0190*	
H4	−0.31010	0.22660	0.22810	0.0190*	
H6	0.25500	0.33730	0.41000	0.0170*	
H7	0.57530	0.42350	0.34770	0.0170*	
H10	1.14920	0.59910	0.08410	0.0200*	
H11	1.38190	0.63530	0.23510	0.0190*	
H13	0.89690	0.49900	0.37330	0.0180*	

### Atomic displacement parameters (Å^2^)

**Table:**

	*U*^11^	*U*^22^	*U*^33^	*U*^12^	*U*^13^	*U*^23^
Br1	0.0189 (1)	0.0213 (1)	0.0161 (1)	−0.0045 (1)	0.0003 (1)	0.0053 (1)
F1	0.0209 (6)	0.0273 (6)	0.0115 (5)	−0.0064 (5)	−0.0028 (4)	0.0011 (4)
O1	0.0208 (7)	0.0184 (6)	0.0119 (6)	−0.0042 (5)	0.0007 (5)	0.0008 (5)
O2	0.0326 (8)	0.0292 (8)	0.0243 (7)	−0.0159 (7)	−0.0022 (6)	−0.0043 (6)
O3	0.0880 (16)	0.0804 (15)	0.0135 (8)	−0.0628 (13)	−0.0074 (9)	0.0082 (8)
N1	0.0128 (7)	0.0141 (7)	0.0150 (7)	0.0001 (6)	0.0013 (6)	−0.0005 (5)
N2	0.0290 (9)	0.0228 (8)	0.0156 (8)	−0.0085 (7)	−0.0011 (7)	−0.0008 (6)
C1	0.0110 (8)	0.0124 (8)	0.0136 (8)	0.0021 (6)	0.0011 (6)	−0.0003 (6)
C2	0.0137 (8)	0.0135 (8)	0.0133 (8)	0.0026 (6)	0.0015 (6)	0.0004 (6)
C3	0.0156 (9)	0.0171 (8)	0.0140 (8)	0.0000 (7)	−0.0011 (6)	−0.0047 (7)
C4	0.0145 (8)	0.0144 (8)	0.0186 (9)	−0.0005 (7)	0.0001 (7)	−0.0018 (7)
C5	0.0144 (8)	0.0148 (8)	0.0127 (8)	0.0009 (7)	0.0015 (6)	0.0021 (6)
C6	0.0147 (8)	0.0160 (8)	0.0125 (8)	0.0014 (7)	0.0000 (6)	0.0002 (6)
C7	0.0123 (8)	0.0151 (8)	0.0137 (8)	0.0012 (7)	−0.0005 (6)	−0.0014 (6)
C8	0.0129 (8)	0.0129 (8)	0.0151 (8)	0.0007 (6)	0.0016 (7)	−0.0003 (6)
C9	0.0139 (8)	0.0179 (8)	0.0125 (8)	0.0022 (7)	−0.0023 (6)	−0.0014 (6)
C10	0.0164 (9)	0.0166 (8)	0.0167 (9)	0.0011 (7)	0.0022 (7)	0.0036 (7)
C11	0.0155 (8)	0.0126 (8)	0.0191 (9)	−0.0009 (7)	0.0017 (7)	0.0007 (7)
C12	0.0163 (9)	0.0155 (8)	0.0145 (8)	−0.0004 (7)	−0.0010 (7)	−0.0021 (6)
C13	0.0160 (9)	0.0159 (8)	0.0143 (8)	−0.0009 (7)	0.0012 (7)	0.0007 (6)

### Geometric parameters (Å, º)

**Table:**

Br1—C5	1.8980 (18)	C5—C6	1.380 (3)
F1—C9	1.339 (2)	C8—C13	1.390 (3)
O1—C2	1.347 (2)	C8—C9	1.399 (2)
O2—N2	1.218 (2)	C9—C10	1.383 (3)
O3—N2	1.215 (3)	C10—C11	1.387 (3)
O1—H1	0.8400	C11—C12	1.383 (3)
N1—C7	1.284 (2)	C12—C13	1.387 (3)
N1—C8	1.409 (2)	C3—H3	0.9500
N2—C12	1.473 (2)	C4—H4	0.9500
C1—C6	1.405 (2)	C6—H6	0.9500
C1—C7	1.453 (2)	C7—H7	0.9500
C1—C2	1.411 (2)	C10—H10	0.9500
C2—C3	1.399 (3)	C11—H11	0.9500
C3—C4	1.376 (3)	C13—H13	0.9500
C4—C5	1.395 (2)		
			
C2—O1—H1	109.00	F1—C9—C10	118.47 (15)
C7—N1—C8	121.86 (16)	C8—C9—C10	123.70 (16)
O2—N2—C12	118.63 (16)	C9—C10—C11	118.36 (16)
O3—N2—C12	118.09 (18)	C10—C11—C12	118.40 (17)
O2—N2—O3	123.29 (18)	N2—C12—C11	118.69 (16)
C2—C1—C7	121.43 (15)	C11—C12—C13	123.40 (17)
C6—C1—C7	119.05 (15)	N2—C12—C13	117.91 (16)
C2—C1—C6	119.52 (16)	C8—C13—C12	118.76 (16)
O1—C2—C3	117.94 (15)	C2—C3—H3	120.00
C1—C2—C3	119.50 (16)	C4—C3—H3	120.00
O1—C2—C1	122.56 (16)	C3—C4—H4	120.00
C2—C3—C4	120.43 (16)	C5—C4—H4	120.00
C3—C4—C5	120.02 (17)	C1—C6—H6	120.00
Br1—C5—C4	119.11 (13)	C5—C6—H6	120.00
Br1—C5—C6	119.98 (13)	N1—C7—H7	120.00
C4—C5—C6	120.88 (16)	C1—C7—H7	120.00
C1—C6—C5	119.65 (15)	C9—C10—H10	121.00
N1—C7—C1	120.45 (16)	C11—C10—H10	121.00
N1—C8—C9	116.27 (16)	C10—C11—H11	121.00
N1—C8—C13	126.35 (16)	C12—C11—H11	121.00
C9—C8—C13	117.38 (16)	C8—C13—H13	121.00
F1—C9—C8	117.83 (16)	C12—C13—H13	121.00
			
C8—N1—C7—C1	−179.92 (16)	C3—C4—C5—C6	0.4 (3)
C7—N1—C8—C9	179.13 (17)	C3—C4—C5—Br1	−177.66 (14)
C7—N1—C8—C13	−1.3 (3)	Br1—C5—C6—C1	177.55 (13)
O3—N2—C12—C13	−4.7 (3)	C4—C5—C6—C1	−0.5 (3)
O2—N2—C12—C11	−3.3 (3)	N1—C8—C9—C10	179.72 (16)
O2—N2—C12—C13	175.80 (17)	C13—C8—C9—F1	−179.08 (16)
O3—N2—C12—C11	176.20 (19)	C13—C8—C9—C10	0.1 (3)
C7—C1—C2—C3	178.71 (17)	N1—C8—C9—F1	0.5 (2)
C7—C1—C6—C5	−178.80 (17)	N1—C8—C13—C12	−179.49 (17)
C2—C1—C7—N1	0.1 (3)	C9—C8—C13—C12	0.1 (3)
C2—C1—C6—C5	0.7 (3)	F1—C9—C10—C11	178.83 (16)
C6—C1—C2—C3	−0.8 (3)	C8—C9—C10—C11	−0.4 (3)
C7—C1—C2—O1	−1.2 (3)	C9—C10—C11—C12	0.4 (3)
C6—C1—C7—N1	179.58 (16)	C10—C11—C12—N2	178.83 (16)
C6—C1—C2—O1	179.35 (16)	C10—C11—C12—C13	−0.3 (3)
O1—C2—C3—C4	−179.43 (16)	N2—C12—C13—C8	−179.08 (16)
C1—C2—C3—C4	0.7 (3)	C11—C12—C13—C8	0.0 (3)
C2—C3—C4—C5	−0.5 (3)		

### Hydrogen-bond geometry (Å, º)

**Table:**

*D*—H···*A*	*D*—H	H···*A*	*D*···*A*	*D*—H···*A*
O1—H1···N1	0.84	1.86	2.601 (2)	146
C7—H7···O3^i^	0.95	2.45	3.399 (3)	173
C13—H13···O3^i^	0.95	2.48	3.430 (3)	173

Symmetry code: (i) −*x*+2, −*y*+1, −*z*+1.
